# MicroRNAs Modulating Cancer Immunotherapy Mechanisms and Therapeutic Synergies

**DOI:** 10.3390/cancers17243978

**Published:** 2025-12-13

**Authors:** Naorem Loya Mangang, Samantha K. Gargasz, Sai Ghanesh Murugan, Munish Kumar, Girish C. Shukla, Sivakumar Vijayaraghavalu

**Affiliations:** 1 Department of Life Sciences (Zoology), Manipur University (A Central University), Imphal 795003, Manipur, India; naoremloya@gmail.com; 2 Center for Gene Regulation in Health and Disease, Department of Biological, Geo and EVS Sciences, 2121 Euclid Ave., Cleveland, OH 44115, USA; s.k.gargasz@vikes.csuohio.edu (S.K.G.); g.shukla@csuohio.edu (G.C.S.); 3 Department of Medical Sciences and Technology, Indian Institute of Technology Madras, Chennai 600036, Tamil Nadu, India; saighanesh2000@gmail.com; 4 Department of Biochemistry, Faculty of Science, University of Allahabad, Prayagraj 211002, Uttar Pradesh, India; munishkp@gmail.com

**Keywords:** microRNA, cancer immunotherapy, immune checkpoints, biomarkers, exosomal miRNAs, tumor microenvironment, immune resistance, miRNA therapeutics, precision oncology

## Abstract

**Simple Summary:**

Immunotherapy has transformed cancer treatment, but many patients do not respond or develop resistance. microRNAs (miRNAs) play a crucial role in regulating the interaction between cancer cells and immune cells. miRNA can turn immune checkpoint signals on or off, alter the behavior of T cells and macrophages, and travel in tiny vesicles between cells to influence the tumor environment. Because of these functions, miRNAs are being studied as biomarkers to predict who will benefit from immunotherapy, and as drugs themselves, either to block harmful miRNAs or replace protective ones. Although the first clinical trials faced safety and delivery challenges, new technologies such as nanoparticles and exosome-based systems are making miRNA therapies more feasible. This review synthesizes the latest evidence on how miRNAs can enhance cancer immunotherapy and explores future directions for translating these findings into clinical practice.

**Abstract:**

Cancer immunotherapy has transformed oncology, but lasting responses are still limited due to resistance mechanisms within the tumor microenvironment. MicroRNAs (miRNAs) have emerged as critical regulators of immune checkpoint pathways, antigen presentation, T-cell activity, and macrophage polarization. By modulating both tumor-intrinsic and immune cell–intrinsic processes, miRNAs influence the efficacy of immune checkpoint inhibitors, therapeutic vaccines, and adoptive cell therapies. Additionally, circulating and exosomal miRNAs are being investigated as minimally invasive biomarkers to predict patient response and resistance to immunotherapy. Clinical trials of miRNA-based treatments, including mimics and inhibitors, have highlighted both the promise and challenges of translating these molecules into clinical use. Advances in delivery systems, RNA chemistry, and combinatorial strategies are paving the way for their integration into precision immuno-oncology. This review offers a comprehensive overview of the mechanistic, biomarker, and therapeutic roles of miRNAs in cancer immunotherapy, highlighting ongoing clinical progress and prospects.

## 1. Introduction

**Figure 1:**
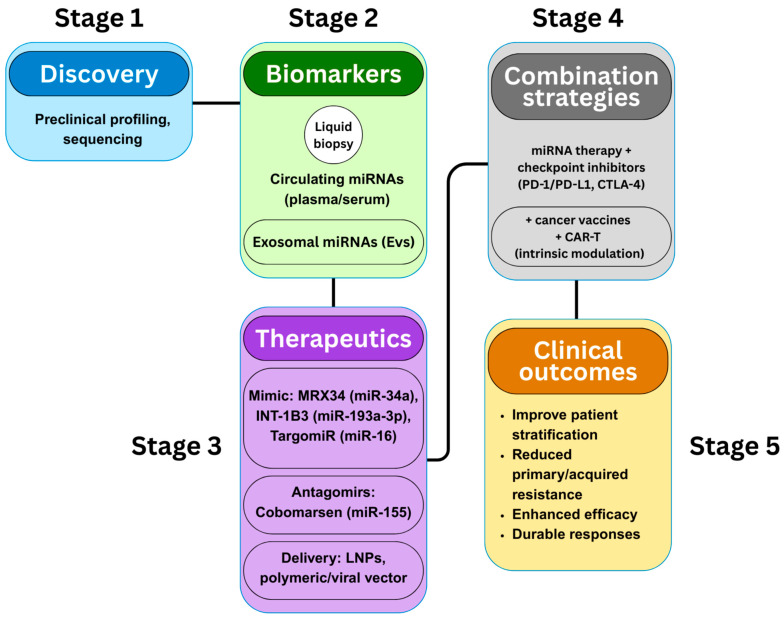
Translational framework illustrates the progression from miRNA discovery and biomarker development to therapeutic formulation, combination with immunotherapies, and ultimate clinical outcomes in cancer immunotherapy.

Cancer immunotherapy has established immune checkpoint inhibitors (ICIs), chimeric antigen receptor (CAR) T-cell therapy, and therapeutic vaccines as key approaches in modern oncology [1,2]. Despite their success in treating melanoma, lung, and hematological malignancies, many patients face primary or acquired resistance, highlighting the urgent need for predictive biomarkers and new combination strategies [3]. MicroRNAs (miRNAs), approximately 22-nucleotide non-coding RNAs that control gene expression post-transcription, have emerged as critical modulators of anti-tumor immunity [4]. Dysregulated miRNA expression contributes to immune escape by changing how tumors interact with immune cells and by shaping the tumor microenvironment (TME), ultimately influencing the efficacy of ICIs [5]. Certain miRNAs directly influence checkpoint molecules like PD-1, PD-L1, and CTLA-4; for instance, miR-200 downregulates PD-L1 and predicts ICI response in non-small-cell lung cancer, while oncogenic miRNAs such as miR-21 promote PD-L1 increase and inhibit PTEN, aiding immune evasion [6,7]. Beyond immune checkpoints, miRNAs also control tumor-associated macrophage polarization, T-cell differentiation, and the recruitment of myeloid-derived suppressor cells, thus directing the immunosuppressive environment [4,8]. MiRNAs carried by extracellular vesicles (EVs) facilitate cell-to-cell communication, reprogramming immune and stromal cells to support tumor growth and resistance to immunotherapy [9,10]. Clinically, circulating miRNA profiles are emerging as minimally invasive biomarkers to predict responses to ICIs and the risk of immune-related adverse events [1,3]. Meanwhile, therapeutic strategies using miRNA mimics, antagomirs, and engineered vesicle delivery systems are being developed to enhance effects when combined with ICIs, vaccines, and CAR-T therapies, offering promising translational prospects [7,9,10,11]. This review summarizes current knowledge about miRNA regulation in cancer immunotherapy, examines therapeutic combinations with checkpoint inhibitors and vaccines, emphasizes future directions in biomarker development and delivery platforms, and outlines a five-stage translational pipeline from discovery to clinical application (Figure 1). Unless otherwise specified, the mechanistic findings summarized in this review are derived from a combination of cell-based, animal, and early translational studies, with the model type highlighted when essential for interpretation.

## 2. Molecular Mechanisms of miRNA-Mediated Immune Regulation

In this section we outline the canonical RISC targeting mechanism, how miRNAs directly and via extracellular vesicles regulate immune checkpoints and co-stimulatory signals, their integration with oncogenic signaling, and cell-type specific roles in T cells, dendritic cells, NK cells and macrophages—ending with a concise synthesis that links mechanisms to therapeutic strategies.

### 2.1. miRNA-RISC Targeting Mechanism

miRNA-mediated immune regulation occurs through sophisticated post-transcriptional control mechanisms centered on the RNA-induced silencing complex (RISC) [12]. Mature miRNAs guide Argonaute proteins to complementary sequences mainly within the 3′ untranslated regions (3′UTRs) of target mRNAs, although functional binding sites can also be found within coding sequences and 5′UTRs. Depending on complementarity and RISC composition, binding causes translational repression or mRNA decay [13]. The canonical targeting mechanism involves the miRNA “seed region” (nucleotides 2–8 from the 5′ end), which shows perfect or near-perfect complementarity to target sites [14]. Recent structural studies demonstrate that supplemental base-pairing beyond the seed region, especially at positions 11–16, improves target specificity and is vital for evolutionarily conserved miRNAs such as let-7 [12,15]. Silencing efficiency increases when multiple RISCs cooperatively bind closely spaced sites (≈15–35 nt), producing synergistic repression. Additionally, “seedless” sites cooperate with canonical binding sites to produce strong target suppression [14]. These basic targeting rules (seed pairing, supplemental pairing and cooperative RISC binding) provide the mechanistic basis for both direct miRNA control of immune checkpoint and co-stimulatory transcripts and for selective miRNA sorting into extracellular vesicles that mediate intercellular reprogramming.

### 2.2. Direct Targeting of Immune Checkpoints and Co-Stimulatory Molecules

miRNAs regulate anti-tumor immunity through both direct post-transcriptional control of immune checkpoints and extracellular vesicle (EV)-mediated communication that reshapes the tumor microenvironment (TME). Several miRNAs directly target immune checkpoint molecules, such as miR-138-5p, which binds the PD-L1 3′UTR to reduce PD-L1 expression and restore CD8^+^ T-cell cytotoxicity in non-small cell lung cancer [16,17,18], and miR-34a, a tumor-suppressive miRNA that represses PD-L1, SYT1 and PDGFRA while influencing macrophage polarization through the p53–miR-34a–CSF1R axis [19,20]; therapeutic delivery of miR-34a using antibody–oligonucleotide conjugates effectively lowers PD-L1, enhances macrophage phagocytosis and increases CD8^+^ T-cell infiltration with minimal systemic toxicity [21,22]. miRNAs also modulate co-stimulatory pathways, exemplified by miR-424, which suppresses CD80 in dendritic cells to disrupt CD80/CTLA-4 interactions, reverse chemoresistance and enhance CD8^+^ T-cell expansion while reducing Treg and myeloid-derived suppressor-cell recruitment [23,24]. In parallel, tumor-derived EVs transport miRNAs that modulate immune and stromal populations, with selective packaging orchestrated by proteins such as hnRNPA2B1 and AGO2 that recognize specific sequence motifs [25,26,27]. Tumor cells commonly export tumor-suppressive miRNAs to discard them or deliver oncomiRs to promote immunosuppressive states [28]. Exosomal miR-21 drives macrophage M2 polarization by targeting KLF3 and activating Nanog/Oct4 signaling in pancreatic neuroendocrine tumors [29,30,31,32], while breast cancer EVs transfer miR-10b to NK cells, downregulating MICB and impairing NK-mediated cytotoxicity; antagomir-mediated inhibition of miR-10b restores NK activation and eliminates metastases in vivo [33,34,35]. Additional EV-associated miRNAs contribute to TME remodeling, including miR-146a, which limits NF-κB–driven inflammation and fibroblast activation by targeting TRAF6, RIPK2 and PTGES2 [36,37,38], and miR-155, which regulates dendritic-cell IL-12 production and antigen presentation by targeting SOCS-1 [39,40,41]. Collectively, these direct and EV-mediated miRNA networks modulate checkpoint expression, reprogram immune cells and reinforce an immunosuppressive TME, driving tumor progression and shaping responses to immunotherapy. Having described how miRNAs act both cell-intrinsically and via EV transfer to reprogram immune and stromal cells, we now consider how oncogenic signaling pathways feed into these miRNA networks and modulate immune escape.

### 2.3. Upstream Signaling Integration

miR-21, one of the most extensively studied oncomiRs, modulates the PTEN/AKT pathway in lung cancer by directly targeting PTEN’s 3′UTR, which leads to activation of the PI3K/AKT pathway and downstream effects on cell proliferation, resistance to apoptosis, metastasis, and resistance to immunotherapy [42,43]. miR-21 promotes lung cancer progression through multiple interconnected signaling networks, including the PI3K/AKT, MEK/ERK, TGF-β/SMAD, Hippo, NF-κB, and STAT3 pathways, while also decreasing sensitivity to chemotherapeutic agents like carboplatin, paclitaxel, cisplatin, and gemcitabine by suppressing PTEN and enhancing DNA repair mechanisms [44,45]. The therapeutic significance of miR-21 goes beyond its direct oncogenic effects; it also contributes to immune evasion, as PTEN loss results in increased PD-L1 expression through PI3K/AKT-mediated transcriptional upregulation [46,47]. Conversely, inhibiting miR-21 restores PTEN function, makes cells more sensitive to radiotherapy, and improves combined treatments involving chemotherapy and immunotherapy [48,49]. These mechanistic insights highlight miRNAs as key regulators that connect multiple oncogenic and immune regulatory pathways, demonstrating their potential as both therapeutic targets and biomarkers for predicting treatment responses across various cancer types and therapeutic approaches. It is important to note that miR-21 may exhibit context-dependent roles, including occasional tumor-suppressive effects under specific cellular or inflammatory conditions [50].

### 2.4. Modulation of the Tumor Microenvironment

**Figure 2:**
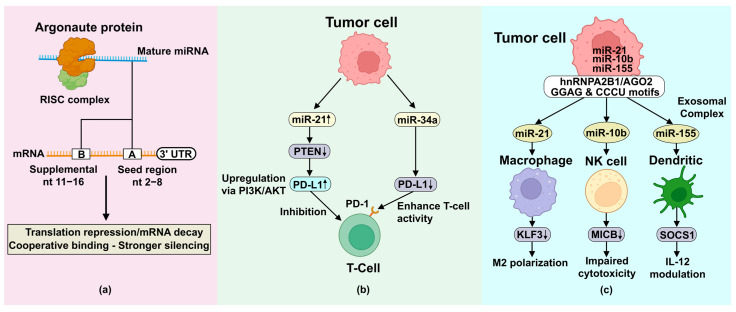
Mechanistic overview of miRNA-mediated regulation of antitumor immunity. (**a**) Canonical RISC targeting. Mature miRNAs are incorporated into the Argonaute (AGO)-containing RISC complex, where the seed region (nt 2–8) and supplemental pairing stabilize binding to target mRNAs at the 3′UTR, resulting in translation repression or mRNA decay. (**b**) Tumor-intrinsic regulation. Within tumor cells, oncogenic miR-21 suppresses PTEN, increasing PD-L1 expression and promoting CD8^+^ T-cell inhibition. Conversely, miR-34a targets the PD-L1 3′UTR, reducing PD-L1 levels and enhancing cytotoxic T-cell activity. (**c**) EV-mediated horizontal transfer. Tumor cells selectively package miRNAs (e.g., miR-21, miR-10b, miR-155) into extracellular vesicles via AGO2/hnRNPA2B1-dependent sorting motifs and release them into the tumor microenvironment. These EV-delivered miRNAs reprogram recipient immune cells: miR-21 induces M2 macrophage polarization, miR-10b reduces MICB expression and impairs NK-cell cytotoxicity, and miR-155 modulates SOCS-1 and IL-12 signaling in dendritic cells. Together, these pathways cooperatively reshape antitumor immunity and promote an immunosuppressive microenvironment.

The tumor microenvironment is a dynamic ecosystem where miRNAs regulate complex intercellular communication networks through extracellular vesicle (EV)-mediated transfer, fundamentally reshaping the immunological landscape and creating either pro-tumorigenic or anti-tumorigenic conditions depending on the specific miRNA cargo and recipient cell populations [25]. miRNA transfer via extracellular vesicles, particularly exosomes ranging from 30–100 nanometers, has become a sophisticated mechanism of horizontal gene transfer that allows cancer cells to reprogram distant and adjacent stromal components, immune effector cells, and other malignant cells within the tumor microenvironment through the selective packaging and delivery of regulatory miRNAs [26,27]. The molecular machinery governing miRNA sorting into EVs involves complex interactions between miRNA sequences, RNA-binding proteins such as hnRNPA2B1, sumoylated hnRNPA2B1, and Argonaute 2 (AGO2), and the endosomal sorting complex required for transport (ESCRT). Specific sequence motifs (GGAG, CCCU) and secondary structures determine the preferential loading of particular miRNAs into exosomal compartments (Figure 2) [51,52,53]. This selective packaging process allows tumor cells to strategically modulate their microenvironment, either by sequestering tumor-suppressive miRNAs into exosomes for export or loading oncogenic miRNAs for delivery to target cells, thereby maintaining cellular homeostasis while influencing surrounding tissue architecture [54].

Immunosuppressive miRNA transfer is exemplified by miR-21 in pancreatic neuroendocrine tumor (PNET) exosomes, which polarizes tumor-associated macrophages toward the immunosuppressive M2 phenotype by directly targeting Krüppel-like factor 3 (KLF3) and activating stemness-promoting transcription factors like Nanog and Oct4 [29,30,31]. Mechanistically, M2 macrophage-derived exosomes rich in miR-21-5p are easily taken up by pancreatic cancer stem cells (CD24+CD44+EpCAM+), where miR-21 binds to the 3′ UTR of KLF3 mRNA, causing its degradation and leading to increased expression of stem cell transcription factors that enhance sphere formation, colony growth, invasion, migration, and resistance to apoptosis [30,31]. In vivo studies demonstrate that pancreatic cancer stem cells co-cultured with miR-21-overexpressing M2 macrophages display significantly higher tumorigenicity, with increased tumor size and weight, and elevated stemness marker levels, while knocking down miR-21 in M2 macrophage-derived exosomes completely prevents these tumor-promoting effects and reduces Nanog/Oct4 levels in recipient cells [30,32]. This reciprocal communication creates a feed-forward loop where PNET cells initially release factors that polarize macrophages toward M2, which then send out miR-21-enriched exosomes that further enhance cancer stem cell properties and treatment resistance, ultimately contributing to the aggressive behavior and poor prognosis of pancreatic neuroendocrine tumors [55].

NK cell suppression via EV-mediated miRNA delivery is another key mechanism of immune evasion. miR-10b in breast cancer EVs directly targets and downregulates stress-induced cell surface molecules, including MICB (MHC class I polypeptide-related sequence B), a critical ligand for NKG2D receptors on natural killer (NK) cells, which are crucial for recognizing and cytotoxic elimination [33,34]. miR-10b, significantly overexpressed in metastatic breast cancer compared to primary tumors, functions as a master regulator of metastasis by promoting epithelial–mesenchymal transition through HOXD10 suppression, increasing invasive capacity by downregulating E-cadherin, and granting stem cell-like properties that enable chemoresistance and immune evasion [35,56]. Extracellular vesicles from triple-negative breast cancer cell lines (MDA-MB-231), which contain elevated miR-10b, effectively transfer this oncomiR to NK cells. This process results in decreased MICB expression on cancer cells, reduced NK cell activation, impaired cytotoxic granule release, and ultimately compromised immune surveillance within the tumor microenvironment [57]. The therapeutic significance of this mechanism is highlighted by studies demonstrating that magnetic nanoparticle-conjugated anti-miR-10b (MN-anti-miR10b) treatment achieves 99% knockdown of miR-10b in primary tumors and metastatic lesions, restores NK cell function, prevents metastasis formation while eliminating existing metastases when combined with conventional chemotherapy in preclinical models [58].

Tumor-suppressive miRNA regulation of stromal components is exemplified by miR-146a, which inhibits tumor-associated fibroblasts (TAFs) in colorectal cancer by targeting multiple inflammatory signaling intermediates, including TRAF6 (TNF receptor-associated factor 6), RIPK2 (receptor-interacting protein kinase 2), and PTGES2 (prostaglandin E synthase 2). This effectively disrupts the IL-17-mediated inflammatory cascade that drives both colitis-associated and sporadic colorectal carcinogenesis [36,59]. miR-146a acts as a master negative regulator of colonic inflammation by simultaneously targeting RIPK2 in myeloid cells to limit the production of IL-17-inducing cytokines (IL-1β, IL-6, IL-23) and TRAF6 in intestinal epithelial cells to restrict responsiveness to IL-17 signaling. Additionally, targeting PTGES2 reduces prostaglandin E2 synthesis, which promotes tumor growth and angiogenesis [37]. The potential of restoring miR-146a has been validated through preclinical studies showing that systemic administration of miR-146a mimics effectively alleviates both DSS-induced colitis and AOM/DSS-driven colorectal cancer. Treated mice exhibit reduced tumor burden, decreased inflammatory cytokine production, lower Ki67 proliferation indices, and significantly improved survival compared to control animals [60]. Mechanistically, miR-146a deficiency leads to enhanced IL-17 signaling, increased infiltration of inflammatory cells, loss of intestinal barrier function, and progression from low-grade adenomas to invasive adenocarcinomas. Restoring miR-146a or pharmacologically inhibiting its targets (TRAF6, RIPK2) reverses these pathological changes and prevents tumor development [38].

Cytokine regulation through miRNA-mediated control of dendritic cell function is exemplified by miR-155, which modulates IL-12 production by directly targeting SOCS-1 (suppressor of cytokine signaling 1), a negative regulator of JAK/STAT signaling that functions as an antigen presentation attenuator by limiting IL-12p40 and IL-12p70 production in mature dendritic cells [39,40]. During human monocyte differentiation into immature dendritic cells and subsequent maturation, miR-155 expression is progressively upregulated. It reaches peak levels in response to TLR/IL-1 inflammatory stimuli, where it forms part of a negative feedback loop that initially promotes IL-12 production by targeting SOCS-1 but subsequently limits excessive inflammatory cytokine release to prevent tissue damage [61]. The functional significance of miR-155 in dendritic cell biology is demonstrated through studies showing that silencing miR-155 in mature dendritic cells results in increased SOCS-1 protein expression, significantly reduced IL-12p70 secretion, a decreased capacity to activate NK cells for IFN-γ production, and an impaired ability to prime Th1 immune responses [62]. Conversely, forced overexpression of miR-155 in immature dendritic cells enhances IL-12p70 production in response to LPS, poly(I:C), and other maturation stimuli, increases the capacity of dendritic cells to activate autologous NK cells for IFN-γ secretion, and improves their functionality as antigen-presenting cells in cancer immunotherapy contexts [63]. These findings emphasize the potential for miR-155-based approaches to enhance dendritic cell vaccine efficacy by optimizing IL-12 production and improving the activation of both innate and adaptive immune responses against tumor antigens; however, miR-155 also exhibits context-dependent behavior and may promote tumorigenesis under chronic inflammatory conditions [41].

The integration of EV-mediated miRNA transfer with tumor microenvironment modulation represents a paradigm shift in understanding cancer progression and therapeutic resistance, revealing opportunities for intercepting these communication networks through targeted miRNA inhibition, exosome engineering, or combination strategies that simultaneously target multiple miRNA-regulated pathways within the complex tumor ecosystem.

### 2.5. Oncogenic miRNAs and Immune Evasion

OncomiRs represent a distinct class of miRNAs promoting checkpoint ligand expression and immune evasion [64]. miR-20b functions as a critical regulator of PD-L1 upregulation in glioblastoma through mechanisms that correlate with treatment resistance and immune suppression [65,66]. In the complex glioblastoma microenvironment, where immune infiltration is naturally limited by the blood–brain barrier and immunosuppressive factors, oncomiRs contribute to the establishment of immune privilege by enhancing checkpoint ligand expression and promoting the accumulation of regulatory T cells, creating formidable barriers to the efficacy of immunotherapy, which requires multimodal therapeutic approaches [67]. The identification of oncomiRs reveals therapeutic opportunities through miRNA inhibition strategies, including antisense oligonucleotides, miRNA sponges, and small molecule inhibitors that can reverse immune evasion phenotypes and sensitize tumors to checkpoint blockade [18].

### 2.6. Context-Specific miRNA Functions

Context-specificity in miRNA function is exemplified by the different roles of hypoxia-induced miR-210 versus normoxic miR-200c in melanoma [28,68]. miR-210 functions as the “hypoxamir,” promoting immune evasion and treatment resistance under low oxygen conditions, while miR-200c acts as a tumor suppressor that enhances drug sensitivity and decreases metastatic potential under normoxic conditions [69,70]. miR-210 is directly upregulated by HIF-1α during hypoxia and promotes cancer stem cell phenotypes, epithelial–mesenchymal transition, and immune suppression by regulating CD24, CD44, CD133, E-cadherin, vimentin, and Snail expression, while simultaneously modulating HIF-1α through negative feedback loops that maintain cellular adaptation to hypoxic stress [71,72]. Conversely, miR-200c shows a decreasing expression pattern from melanocytic nevi to primary melanomas and metastatic lesions, and restoring it significantly reduces cell proliferation, migration, and drug resistance by downregulating BMI-1, ABCG2, ABCG5, and MDR1, while increasing E-cadherin expression, effectively reversing aggressive melanoma phenotypes [73,74]. The opposing roles of these miRNAs in response to microenvironmental conditions highlight the importance of considering tumor oxygenation and metabolic context when developing miRNA-based therapies [75].

### 2.7. miRNA Regulation of Immune Cell Activation

The orchestration of immune cell activation represents one of the most sophisticated regulatory networks in human biology, where miRNAs function as master conductors that fine-tune the development, differentiation, and effector functions of both innate and adaptive immune cells through precisely timed expression programs and context-dependent targeting mechanisms [76,77]. These small regulatory RNAs control critical checkpoints in immune cell maturation, activation thresholds, cytokine production, and memory formation, ultimately determining the magnitude, duration, and specificity of immune responses in both physiological and pathological contexts, including cancer immunotherapy [78,79,80].

#### 2.7.1. miRNAs in T-Cell Differentiation

The miR-17-92 cluster emerges as a central regulator of CD8^+^ T-cell effector functions, promoting proliferation and terminal effector differentiation through the upregulation of PI3K-AKT-mTOR signaling pathways [81,82]. This polycistronic miRNA cluster, encoding six individual miRNAs (miR-17, miR-18a, miR-19a, miR-20a, miR-19b, and miR-92a), exhibits dynamic temporal expression patterns during CD8^+^ T-cell responses, with peak expression occurring during the rapid proliferation phase of effector expansion and subsequent downregulation as cells transition toward memory phenotypes [83]. Mechanistically, miR-17-92 promotes effector differentiation by targeting multiple tumor suppressors, including PTEN, thereby relieving the inhibition of the PI3K-AKT-mTOR axis and driving metabolic reprogramming toward glycolysis and protein synthesis, which are required for rapid cell division and cytotoxic effector functions [84]. Conditional overexpression of miR-17~92 in CD8^+^ T cells results in enhanced BrdU incorporation, increased proliferation during primary expansion, and preferential differentiation toward short-lived terminal effector cells at the expense of memory precursor cells, while also promoting expression of granzyme B, perforin, and IFN-γ production [85]. Sustained miR-17~92 expression prevents proper memory formation and leads to gradual loss of memory cells over time, providing critical knowledge on the importance of temporal regulation in balancing effector function with long-term protective immunity [86]. From a therapeutic perspective, engineering tumor antigen-specific CD8^+^ T cells to transiently overexpress miR-17-92 enhances their type-1 effector functions, including IFN-γ and TNF-α production, improves cytotoxic activity against target cells, and increases their capacity to control tumor growth in adoptive transfer models, suggesting potential applications in CAR-T cell therapy and tumor-infiltrating lymphocyte expansion protocols [87].

#### 2.7.2. DC Maturation and Antigen Presentation

miR-155 enhancement of dendritic cell function represents a paradigm for leveraging miRNA biology to improve vaccine efficacy, particularly in the context of mRNA vaccines where enhanced antigen presentation is critical for robust immune responses [88]. During dendritic cell maturation, miR-155 expression is progressively upregulated in response to pathogen-associated molecular patterns (PAMPs) and inflammatory cytokines, where it functions as a master regulator of DC activation by targeting multiple negative regulators, including SOCS-1 (suppressor of cytokine signaling 1), c-Fos, Arg-2, and Jarid2 [89,90,91]. The functional consequences of miR-155 upregulation in DCs include enhanced expression of co-stimulatory molecules (CD80, CD86, and CD40), increased MHC class II presentation, improved migration toward lymph nodes via CCR7 upregulation, and augmented IL-12p70 production, which promotes Th1 polarization and NK cell activation [92,93]. Transgenic overexpression of miR-155 in bone marrow-derived dendritic cells results in superior antigen processing and presentation capabilities, enhanced T cell priming capacity, and improved therapeutic efficacy when used as DC vaccines against established breast cancer tumors, with treated mice exhibiting reduced primary tumor growth, dramatically suppressed lung metastasis, and increased effector T cell infiltration [94,95,96]. In the context of mRNA vaccines, miR-155 enhancement could potentially address the challenge of suboptimal antigen presentation by professional antigen-presenting cells, as host miRNAs may interfere with mRNA vaccine translation and reduce antigen production, thereby weakening the resulting immune response [97]. Recent advances have demonstrated that miR-155-enriched tumor-derived exosomes can be used to prime dendritic cells ex vivo, creating “educated” DCs with enhanced immunostimulatory properties that produce superior antitumor responses compared to conventional DC vaccines [98]. The therapeutic application of miR-155 enhancement in DC vaccines represents a promising strategy for improving immunotherapy outcomes, particularly when combined with other immune adjuvants such as TLR ligands, immune-stimulating cytokines, or checkpoint inhibitors [99].

#### 2.7.3. NK Cell Activity Modulation

The miR-122 targeting of killer immunoglobulin-like receptors (KIRs) in natural killer cells represents a sophisticated mechanism for fine-tuning NK cell activation thresholds and cytotoxic capacity, particularly in the context of liver cancer, where miR-122 functions normally as a hepatocyte-specific tumor suppressor but may also regulate infiltrating immune cells [100,101]. miR-122, which constitutes approximately 70% of the total miRNA population in healthy hepatocytes, is significantly downregulated in about 70% of hepatocellular carcinoma cases, contributing to tumor progression, immune evasion, and treatment resistance through loss of its tumor-suppressive functions [102,103]. In NK cells infiltrating the liver, miR-122 potentially modulates KIR expression, which normally provides inhibitory signals when engaged by MHC class I molecules on target cells, thereby preventing NK cell activation against healthy autologous cells while preserving their ability to eliminate malignant or infected cells that have downregulated MHC class I expression [104]. The therapeutic restoration of miR-122 through various delivery systems, including adeno-associated virus vectors (AAV8) and lipid nanoparticles, has demonstrated significant efficacy in reducing hepatocellular carcinoma tumor growth and improving treatment outcomes in preclinical models, potentially through a combination of direct tumor suppression and enhancement of NK cell-mediated immune surveillance [105,106]. Recent studies have also identified miR-122-5p as a critical regulator of decidual NK (dNK) cell function during pregnancy, where it controls trophoblast invasion and vascular remodeling through targeting of multiple genes involved in cell migration, angiogenesis, and immune tolerance, suggesting broader roles for miR-122 in NK cell biology beyond liver cancer [100,107]. The development of miR-122 replacement therapies using advanced delivery platforms could potentially enhance both direct tumor suppression and NK cell-mediated antitumor immunity, creating synergistic therapeutic effects that address multiple aspects of hepatocellular carcinoma pathogenesis [101].

#### 2.7.4. Macrophage Polarization Control

miR-145 promotion of M1 phenotype in lung cancer represents a critical mechanism for shifting the tumor-associated macrophage population from immunosuppressive M2 polarization toward pro-inflammatory, tumoricidal M1 activation which supports antitumor immunity [44,108]. miR-145, along with miR-130a, functions as a key regulator of myeloid cell reprogramming by targeting transforming growth factor-β receptor II (TβRII) and insulin-like growth factor 1 receptor (IGF1R), both of which are critical mediators of immunosuppressive signaling within the tumor microenvironment [109,110]. Mechanistically, miR-145 disrupts TGF-β signaling cascades that usually promote M2 polarization, angiogenesis, and metastatic progression, while simultaneously inhibiting IGF1R-mediated survival and proliferation signals that support tumor-associated macrophage accumulation [111]. Ectopic expression of miR-145 in Gr-1+CD11b+ myeloid cells effectively reprograms them from a pro-tumorigenic to anti-tumorigenic phenotype, resulting in increased production of pro-inflammatory cytokines (IL-1β, TNF-α, IL-6), enhanced nitric oxide synthase activity, and improved antigen presentation capabilities [112]. In preclinical models of breast cancer metastasis, mice receiving miR-145-engineered hematopoietic stem/progenitor cells exhibited significantly reduced lung metastasis without affecting primary tumor size, demonstrating that miR-145-mediated myeloid cell reprogramming specifically targets the metastatic microenvironment [113]. The therapeutic potential of miR-145 extends beyond direct macrophage polarization to encompass effects on other myeloid-derived suppressor cells and regulatory cell populations, resulting in a comprehensive shift in the immune microenvironment that favors tumor elimination over immune evasion [44]. Recent studies have also demonstrated that miR-145 can be delivered through various nanoparticle platforms and combined with IGF1R inhibitors such as NT157 to achieve synergistic effects on tumor metastasis suppression [109].

#### 2.7.5. Context-Specific Roles in Innate vs. Adaptive Immunity

The context-specific functions of miRNAs in innate versus adaptive immunity reflect the evolutionary sophistication of immune regulation, where the same miRNA can exert opposing effects depending on the cell type, activation state, pathogen context, and tissue environment [114]. In innate immunity, miRNAs primarily regulate rapid response mechanisms, including pathogen recognition, inflammatory cytokine production, and antimicrobial effector functions. MiRNAs such as miR-155, miR-146a, and miR-21 serve as critical regulators of TLR signaling, NF-κB activation, and type I interferon responses [115]. miR-155, for example, promotes M1 macrophage activation and inflammatory cytokine production in response to bacterial lipopolysaccharide by targeting SOCS-1 and other negative regulators, while simultaneously enhancing dendritic cell maturation and IL-12 production, which are required for Th1 priming [116]. In certain contexts, such as hepatitis B virus-associated hepatocellular carcinoma, miR-155 paradoxically promotes M2 macrophage polarization through the miR-155/SHIP1 axis, demonstrating how pathogen-specific factors and tissue environments can reverse the typical functions of miRNAs [117]. In adaptive immunity, miRNAs orchestrate more complex developmental programs including T cell differentiation, B cell maturation, antibody class switching, and memory formation, where precise temporal control is essential for balancing effector function with long-term protective immunity [118]. The miR-17-92 cluster exemplifies this complexity by promoting rapid CD8^+^ T cell expansion and effector differentiation during acute infections, while requiring subsequent downregulation to enable proper memory cell formation. This process accentuates how the same miRNA network must be dynamically regulated to serve different phases of the adaptive immune response [119]. miR-29 family members exhibit additional context-specific functions by regulating both innate and adaptive immunity through the targeting of distinct gene networks in various cell types, including the promotion of IFN-γ production in T cells, regulation of antibody production in B cells, and modulation of inflammatory cytokine release in macrophages [120]. The clinical implications of these context-specific miRNA functions are profound for cancer immunotherapy, where therapeutic interventions must account for the complex interplay between innate and adaptive immunity, the heterogeneity of tumor microenvironments, and the potential for miRNA-based therapies to have unintended consequences in different cellular contexts [115]. Future therapeutic strategies will likely require sophisticated delivery systems that can target specific cell types and activation states, temporal control mechanisms that modulate miRNA activity during different phases of immune responses, and combination approaches that address the multifaceted roles of miRNAs in both innate and adaptive immunity [119,120].

## 3. Therapeutic Synergies of miRNAs in Cancer Immunotherapy

### 3.1. miRNA Mimics and Inhibitors as Immunotherapy Enhancers

For miRNAs such as miR-34a and miR-155, whose mechanistic roles were discussed earlier, this section focuses on their therapeutic synergy without repeating prior mechanistic details. Therapeutic strategies that combine synthetic miRNA mimics or inhibitors with immune checkpoint blockade have demonstrated remarkable synergy in preclinical cancer models by concurrently targeting tumor-intrinsic oncogenic pathways and reshaping the immune microenvironment to overcome resistance [18,121]. For example, the systemic delivery of miR-34a mimics encapsulated in lipid nanoparticles downregulates PD-L1 and oncogenic targets such as BCL2 and MET in triple-negative breast cancer, leading to enhanced CD8^+^ T-cell infiltration and a 70% greater tumor reduction when combined with anti-PD-1 therapy compared to monotherapy in murine models [122]. Similarly, exosome-mimetic nanovesicles carrying miR-200c sensitize lung adenocarcinoma to anti-CTLA-4 by targeting ZEB1, thereby restoring E-cadherin expression and promoting dendritic cell maturation and antigen cross-presentation, which yields complete tumor regression in 40% of treated mice and long-term immune memory upon rechallenge [123]. In microsatellite-stable colorectal cancer, where high miR-21 expression mediates resistance to PD-1 blockade via PTEN suppression and PI3K-AKT–driven PD-L1 upregulation, anti-miR-21 oligonucleotides restore PTEN, reduce PD-L1 levels, and enhance CD8^+^ T-cell cytotoxicity, achieving a 60% decrease in tumor burden when combined with anti-PD-1 therapy [124]. Mechanistically, these miRNA-based interventions act through “double-hit” mechanisms: tumor-suppressive mimics directly inhibit checkpoint ligands and oncogenes, while anti-oncomiRs release tumor suppressors and decrease immunosuppressive cytokine production, together converting “cold” tumor microenvironments into immune-permissive states [1]. The translational success of these approaches will depend on advances in delivery platforms—such as targeted lipid nanoparticles, exosome-based carriers, and antibody–miRNA conjugates—that ensure tumor-specific uptake, controlled release kinetics, and minimal off-target activity, thereby maximizing therapeutic efficacy and safety in future clinical applications [125].

### 3.2. Synergy with Checkpoint Inhibitors and mRNA Vaccines

#### 3.2.1. Enhancement of Immune Checkpoint Inhibitor Efficacy

The strategic integration of miRNAs with immune checkpoint inhibitors has demonstrated remarkable therapeutic synergy through complementary mechanisms that enhance antigen presentation, restore immune surveillance, and overcome resistance pathways limiting single-modality approaches [18]. miR-138-5p exemplifies miRNA-mediated enhancement of checkpoint inhibitor efficacy by directly targeting the 3′ untranslated region of PD-L1 (CD274) mRNA, resulting in a 67% reduction in luciferase reporter activity and significant downregulation of PD-L1 expression in A549 and Calu-6 adenocarcinoma cell lines [16]. This tumor-suppressive miRNA simultaneously prevents T-cell exhaustion by reducing surface PD-1 expression on Jurkat cells co-cultured under tumor microenvironment-mimicking conditions with inflammatory cytokines [122]. When combined with anti-PD-1 therapy in preclinical lung cancer models, miR-138-5p and miR-200c demonstrated remarkable efficacy in preventing both benzo(a)pyrene-induced lung adenomas and N-nitroso-tris-chloroethylurea-induced squamous cell carcinomas without detectable systemic toxicity [126]. Single-cell RNA sequencing and imaging mass cytometry revealed that both miRNAs inhibited PD-L1 expression across tumor cell populations while increasing infiltration of CD4^+^ and CD8^+^ T cells and reducing the number of regulatory T cells [127]. The mechanistic basis involves the dual action of miR-138-5p, which directly suppresses checkpoint ligands on tumor cells while simultaneously modulating immune cell activation states, creating a “double-hit” effect that amplifies checkpoint blockade efficacy beyond what either intervention achieves alone [6].

#### 3.2.2. mRNA Vaccine Synergy Enhancement

miRNA-enhanced mRNA vaccine efficacy has been demonstrated through miR-155-mediated dendritic cell optimization, where the overexpression of this master regulatory miRNA significantly improves vaccine-induced antitumor immunity in melanoma and breast cancer models [92,94]. miR-155 functions as a critical regulator of dendritic cell maturation by targeting negative regulators, including SOCS-1, c-Fos, Arg-2, and Jarid2 [41]. This targeting leads to enhanced expression of co-stimulatory molecules (CD80, CD86, CD40), increased MHC class II presentation, improved migration toward lymph nodes via CCR7 upregulation, and augmented IL-12p70 production promoting Th1 polarization [98]. In transgenic mice overexpressing miR-155, dendritic cell vaccines pulsed with tumor antigens resulted in enhanced T-cell priming capacity, increased effector T-cell tumor infiltration, suppressed primary tumor growth, and significantly reduced lung metastasis compared to wild-type DC vaccines [94]. The therapeutic potential of miR-155 enhancement is particularly relevant given recent advances demonstrating that host miRNAs can interfere with mRNA vaccine translation and reduce antigen production, thereby weakening immune responses [96]. By boosting miR-155 expression in dendritic cells, the efficacy of mRNA vaccines could be significantly improved, addressing the suboptimal antigen presentation that currently limits vaccine-driven antitumor immunity [95].

#### 3.2.3. Dual Checkpoint Inhibition Combinations

Combination strategies using dual immune checkpoint inhibitors with miRNA mimics have shown exceptional promise in overcoming resistance to single-agent therapies, especially in immune-cold tumors with low mutational burden [128]. The bispecific antibody cadonilimab (AK104), which targets PD-1 and CTLA-4 simultaneously while lacking an Fc region to prevent inflammatory cytokine secretion, has demonstrated favorable safety profiles and encouraging antitumor activity in cervical cancer, NSCLC, and hepatocellular carcinoma [129]. Preclinical studies combining miR-200c mimics with dual anti-PD-1/CTLA-4 blockade achieved tumor regression rates exceeding 60% in lung adenocarcinoma models, compared to 25% with dual checkpoint inhibition alone, while also inducing robust immunological memory that protects against tumor rechallenge [130]. The mechanistic rationale involves miRNA-driven restoration of immune checkpoint sensitivity through direct targeting of resistance pathways, improved antigen presentation via dendritic cell activation, and reversal of immunosuppressive tumor microenvironments, creating synergistic effects that overcome the limitations of individual therapies [131].

### 3.3. Advanced Delivery Systems for miRNA Therapeutics

The clinical application of miRNA-based cancer immunotherapy has been fundamentally limited by delivery challenges, leading to extensive research into sophisticated nanocarrier platforms that can overcome miRNAs’ inherent instability, improve tumor targeting accuracy, and reduce off-target effects while preserving therapeutic potency [132]. Nanoparticle-based delivery systems have emerged as the most extensively studied platforms for miRNA therapeutics, with lipid nanoparticles (LNPs) showing great promise for miR-34 a mimics because they can protect miRNAs from ribonuclease degradation and enable efficient cellular uptake and controlled intracellular release [133]. Advanced ionizable lipid-based LNPs that use components such as DODMA (1,2-dioleyloxy-3-dimethylaminopropane) and DLin-MC 3-DMA feature pH-responsive charge switching, allowing for effective miRNA encapsulation at low pH during formulation, endosomal escape upon cellular entry, and minimal toxicity under physiological conditions. This addresses key limitations of earlier cationic lipid formulations that experienced protein aggregation and hemolytic activity [134]. Recent innovations in delivering miR-34 a include chitosan-PLGA nanoparticles with hydrodynamic diameters around 139 nm, which show improved cellular distribution, stability, and encapsulation efficiency of 80–100%. These systems also lead to significant upregulation of p53 and downregulation of SIRT 1 in non-small cell lung cancer models, providing valuable insight into the therapeutic potential of hybrid polymeric systems that blend biodegradability with controlled release kinetics [135]. The effectiveness of nanoparticle-mediated miR-34 a delivery has been confirmed across various cancer types, including the development of folic acid/protamine/miR-34 a/protamine @ nanodiamond nanohybrids (FA/PS/miR-34 a/PS @ NDs) with a 210 nm diameter and −25 mV zeta potential. These targeted the folate receptor on triple-negative breast cancer cells via endocytosis and showed significant anti-tumor effects by inducing apoptosis, inhibiting proliferation, and preventing migration through targeting Activator protein 1 (AP-1) transcription factors [136,137].

**Figure 3:**
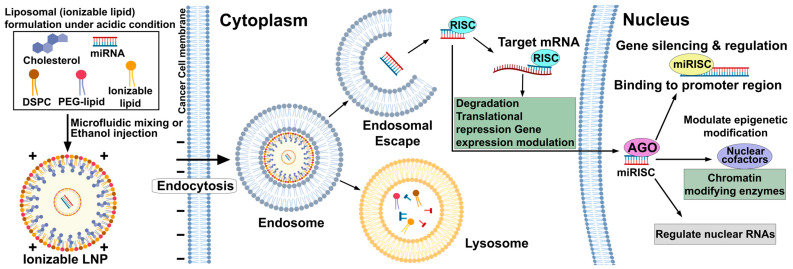
The formulation, cellular uptake and functional mechanisms of miRNA-loaded lipid nanoparticles (LNPs) in cancer therapy. Ionizable LNPs are prepared under acidic conditions using microfluidic mixing or ethanol injection, typically comprising ionizable lipids, helper lipids (DSPC and cholesterol), and PEG-lipids for stability. Following systemic administration, the LNPs are internalized by cancer cells through endocytosis and subsequently escape from endosomes into the cytoplasm, where the released miRNA associates with the RNA-induced silencing complex (RISC) to mediate mRNA degradation, translational repression, and gene expression modulation. A fraction of the miRNA–Argonaute (AGO) complex translocates into the nucleus, where it binds to promoter regions to regulate gene transcription or interacts with chromatin-modifying enzymes and nuclear cofactors to modulate epigenetic states.

Exosome-based delivery systems represent a paradigm shift toward biomimetic nanocarriers that leverage the natural intercellular communication mechanisms of extracellular vesicles to achieve superior biocompatibility, reduced immunogenicity, and enhanced tumor targeting compared to synthetic nanoparticles (Figure 3) [138,139]. Mesenchymal stem cell-derived extracellular vesicles (MSC-EVs) loaded with therapeutic miRNAs, including tumor-derived exosomes carrying miR-146 a, have demonstrated remarkable therapeutic potential through their intrinsic tumor-targeting abilities and capacity for controlled miRNA release within the tumor microenvironment [140,141]. The clinical relevance of miR-146 a delivery via exosomes has been established through studies demonstrating that MSC-derived exosomes engineered to overexpress miR-146 a achieve 67% reduction in tumor weight in ovarian cancer models [142], while mechanistic analyses reveal dual anti-angiogenic effects through direct inhibition of endothelial tube formation (54% reduction) and indirect effects via reduced SERPINE 1 secretion from tumor cells, creating comprehensive anti-tumor activity that targets both cancer cells and supporting vasculature [143]. The therapeutic advantages of exosome-mediated delivery are exemplified by enhanced plant-derived vesicles engineered for improved xenograft penetration and oncolytic effect (HEXPO), which efficiently deliver miR-146 a-5p to the tumor microenvironment and achieve robust tumor growth inhibition through targeting of angiogenesis-related pathways including negative regulation of blood vessel formation, while maintaining excellent biocompatibility and avoiding the immune activation associated with synthetic delivery systems [144]. Importantly, the clinical application of MSC-derived exosomes carrying therapeutic miRNAs has been further validated through studies showing that miR-34 c-overexpressing MSC exosomes with approximately 100 nm particle size effectively attenuate nasopharyngeal carcinoma progression and enhance radiation-induced apoptosis by targeting β-catenin [145,146], demonstrating the potential for combining exosome-mediated miRNA delivery with conventional radiotherapy to overcome treatment resistance [147].

**Figure 4:**
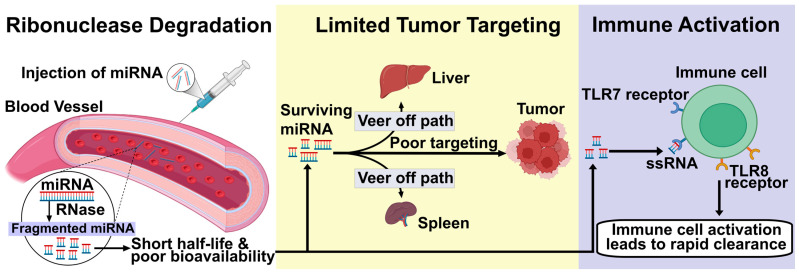
The major biological barriers that limit the efficacy of unmodified miRNA delivery in cancer therapy. Following intravenous injection, miRNA molecules are rapidly degraded by circulating ribonucleases, resulting in fragmented RNA and reduced bioavailability. The small fraction of surviving miRNAs exhibits poor tumor accumulation due to nonspecific biodistribution to off-target organs such as the liver and spleen. Free miRNAs can activate Toll-like receptors (TLR7 and TLR8) on immune cells, leading to immune stimulation and accelerated systemic clearance.

Delivery challenges include three key areas that must be addressed for successful clinical translation: stability issues caused by rapid miRNA degradation by ubiquitous ribonucleases in circulation, resulting in short half-lives and poor bioavailability (Figure 4) [4,148]; limited tumor targeting specificity due to insufficient accumulation at tumor sites and poor cellular uptake by cancer cells [149,150]; and immune activation triggered by recognition of delivery vehicles or miRNA cargo by pattern recognition receptors, which can reduce efficacy and cause adverse effects [99,151]. The stability issue has been partially mitigated through chemical modifications such as 2′-O-methyl, 2′-fluoro, and phosphorothioate modifications that improve nuclease resistance while maintaining miRNA function, though these changes can affect target specificity and require careful optimization to ensure therapeutic effectiveness [152]. Tumor targeting limitations arise from the heterogeneous nature of tumor vasculature, the variable enhanced permeability and retention (EPR) effect across different cancer types, and the necessity for active targeting strategies that can bypass biological barriers, such as the blood–brain barrier, for central nervous system tumors [153,154]. Immune activation presents a dual challenge: delivery systems must avoid triggering innate immune responses that lead to rapid clearance, while also potentially leveraging controlled immune activation to boost anti-tumor immunity. Achieving this balance requires advanced engineering of delivery vehicles to optimize both immune evasion and therapeutic benefit [155].

Advanced delivery innovations have focused on developing stimuli-responsive systems that can overcome traditional delivery limitations through precisely controlled cargo release mechanisms [148]. pH-sensitive nanoparticles utilize the acidic tumor microenvironment (pH 6.0–6.5) to trigger miRNA release specifically at tumor sites, using weakly acidic polymers or lipids that undergo protonation-induced conformational changes to release their therapeutic payload while minimizing systemic exposure to healthy tissues [156]. These systems have been exemplified by PEG-shedding nanoparticles encapsulating both chemotherapeutics and miR-200 for colorectal cancer treatment, demonstrating improved therapeutic effectiveness through site-specific release and reduced off-target toxicity [152]. Aptamer-guided delivery is the most advanced targeting approach, employing single-stranded oligonucleotides that bind with high specificity to overexpressed cell surface receptors on cancer cells, enabling precise delivery of miRNA cargo with minimal off-target effects [157]. The clinical potential of aptamer-mediated miRNA delivery has been shown in studies using GL 21. T and Gint 4. T aptamers that specifically recognize PDGFRα and PDGFRβ, respectively, to deliver anti-miR-222, miR-137, and anti-miR-10 b to glioblastoma cells, achieving receptor-dependent selective modulation of endogenous miRNA levels, increased sensitivity to temozolomide, and inhibition of tumor growth and migration both in vitro and in vivo [4]. The versatility of aptamer-guided delivery is further demonstrated by two-component stick-based methods that allow conjugation of multiple anti-miR sequences (GL 21. T-10 b-222) to single aptamer carriers, providing combination therapeutic effects through targeting multiple oncogenic miRNAs simultaneously while maintaining the structural integrity and binding specificity of both the aptamer and miRNA components [149]. Despite their high specificity and therapeutic potential, aptamer-based delivery systems face clinical translation challenges, including nuclease degradation, high production costs, and the need for better tissue penetration, though ongoing improvements in chemical modifications, in vivo SELEX techniques, and integration with nanoparticle platforms are addressing these issues and moving toward clinical use [158]. The integration of these advanced delivery methods promises to overcome the main barriers limiting miRNA therapeutics, enabling precise, efficient, and safe delivery of therapeutic miRNAs to improve cancer immunotherapy outcomes across various tumor types and clinical scenarios [148,152].

### 3.4. Integration with CAR T-Cell Therapies

The integration of microRNA engineering with CAR T-cell therapy represents a pioneering approach to overcome ongoing challenges such as T-cell exhaustion, immunosuppressive tumor microenvironments, and limited persistence, which have restricted the effectiveness of engineered T cells against solid tumors [159,160]. miRNAs that enhance CAR T-cell persistence have been most thoroughly studied with the miR-17~92 cluster in glioblastoma, where this polycistronic miRNA cluster promotes Th 1 phenotype differentiation, boosts cytotoxic effector functions, and significantly prolongs long-term tumor control capabilities [161,162]. In glioblastoma patients, CD4^+^ T cells are typically polarized toward an unfavorable Th 2 phenotype with decreased miR-17-92 expression—a process further worsened by lymphocytes secreting IL-4, which suppresses miR-17-92 expression and lowers the Th 1/Th 2 ratio, an unfavorable prognosis factor for patient survival [163]. Ohno et al. addressed this issue by engineering CAR T cells with additional miR-17-92 transgene expression using a lentiviral vector (FG 12-EF 1 a-miR-17-92) containing dual promoters: EF 1 a controlling miR-17-92 expression and UbiC controlling EGFP expression for assessing transduction efficiency [160]. In preclinical studies, CAR T cells co-transduced with miR-17-92 (3 C + miR) showed superior long-term stability and greater resistance to temozolomide-induced T-cell suppression compared to standard CAR T cells [161]. Notably, when immunocompromised mice were re-challenged with glioblastoma cells 49 days after initial CAR T-cell infusion, all four mice treated with conventional CAR T cells developed tumors, whereas none of the three mice receiving miR-17-92-enhanced CAR T cells exhibited tumor growth-highlighting the significant impact of miRNA engineering on immunological memory and lasting tumor control [160]. The underlying mechanism of this improved efficacy involves increased CAR T-cell survival, heightened interferon-γ secretion, and the maintenance of anti-tumor activity through the promotion of memory T-cell formation via miR-17-92-mediated targeting of pro-apoptotic and exhaustion-related pathways [162,164].

Overcoming tumor microenvironment suppression through miR-155 targeting TGF-β pathways is a sophisticated strategy to make CAR T cells resistant to immunosuppressive signals while boosting their effector functions within hostile tumor environments [165,166]. miR-155 prevents CD8^+^ T-cell senescence and exhaustion by epigenetically suppressing terminal differentiation factors, achieved by indirectly increasing Polycomb repressor complex 2 (PRC2) activity through promoting Phf19 expression and decreasing Ship1 levels, which inhibit Akt signaling [167]. This pathway is especially important in TGF-β-rich tumor microenvironments, where this cytokine promotes regulatory T-cell differentiation, inhibits effector T-cell functions, and acts as a physical barrier to T-cell infiltration by increasing collagen deposition and stromal remodeling [168]. Recent advances include engineering CAR T cells with chimeric switch receptors that convert inhibitory TGF-β signals into activating ones, along with secreting TGF-β traps that neutralize local TGF-β levels, creating a dual mechanism to resist and overcome tumor immunosuppression [169]. In preclinical solid tumor models, CAR T cells with TGF-β resistance showed significantly better tumor infiltration, sustained proliferation within the tumor microenvironment, and enhanced cytotoxic activity against target cells, while avoiding the T-cell dysfunction usually caused by chronic TGF-β exposure [166,170]. The clinical importance of boosting miR-155 levels in CAR T cells goes beyond TGF-β resistance, as miR-155^−/−^ CD8^+^ T cells in tumor tissues display reduced proliferation and invasion capabilities, which can be restored with immune checkpoint antibody treatment, indicating that miR-155 regulates pathways essential for tumor immune responses [165,167].

Preclinical models utilizing miRNA-modified CAR T cells in sarcomas have shown strong evidence for this approach’s potential to treat aggressive mesenchymal cancers that are often resistant to traditional immunotherapies [171]. New fourth-generation-like CAR miR cells have been created to release therapeutic miRNAs through exosomes while also targeting tumor antigens, such as IL-13(E12Y) CAR constructs that include precursor miR-34a under a 6xNFAT-IL2 minimal promoter activated after CAR-antigen engagement [172]. These engineered CAR miR cells demonstrate significant upregulation and exportation of miR-34a-5p in exosomes, creating a localized therapeutic miRNA delivery system that improves cytotoxic effects against glioblastoma and sarcoma cell lines compared to standard CAR T cells [173]. Their dual role—in direct tumor cell killing via CAR-mediated cytotoxicity and in indirect tumor inhibition through miRNA delivery—marks a promising direction toward multifunctional therapeutic T cells that can address tumor heterogeneity and resistance mechanisms at once [174]. In sarcoma models, miRNA-modified CAR T cells show promise because specific miRNAs like miR-34a can target multiple oncogenic pathways, including restoring p53, causing cell cycle arrest, and triggering apoptosis, while the CAR component ensures targeting of sarcoma-associated antigens like GD2, HER2, or tumor-specific neoantigens [175]. The production of these advanced CAR miR cells involves lentiviral transduction of T cells with constructs encoding both the CAR and miRNA, followed by expansion under optimized conditions that promote a central memory T-cell phenotype, which offers better persistence and anti-tumor activity in solid tumor settings [176]. Clinical translation faces unique hurdles, such as the diverse nature of sarcoma subtypes, limited expression of universal target antigens, and the dense stromal microenvironment characteristic of these tumors, necessitating combination strategies that incorporate chemotherapy or radiation to improve tumor penetration and miRNA delivery [177]. Nonetheless, early preclinical data suggest that miRNA-enhanced CAR T cells could overcome many barriers that have hindered successful CAR T therapy in solid tumors—like poor persistence, limited tumor infiltration, and resistance from immunosuppressive microenvironments, making this a promising next-generation cellular immunotherapy for patients with advanced sarcomas and other solid cancers [178].

### 3.5. Clinical Trials of miRNA-Based Therapeutics in Cancer Immunotherapy

The clinical development of miRNA-based therapeutics has provided important insights into their potential for modulating immune responses in cancer [179,180]. The liposomal miR-34a mimic MRX34 was the first miRNA drug to reach clinical testing (NCT01829971), designed to restore tumor-suppressive activity and sensitize tumors to immune attack; however, the trial was terminated due to immune-mediated toxicities, offering valuable information on safety and delivery challenges [181,182]. Subsequent efforts explored TargomiR, a miR-16 mimic encapsulated in bacterial minicells, which demonstrated activity in malignant pleural mesothelioma (NCT02369198) [183], and Cobomarsen (MRG-106), an antisense inhibitor of the oncogenic miR-155, evaluated in cutaneous T-cell lymphoma and other hematological cancers (NCT02580552; NCT03713320), although its development was halted for non-safety-related business reasons [184,185]. More recently, INT-1B3, a lipid nanoparticle-formulated mimic of miR-193a-3p, entered early-phase testing for solid tumors and represents a new generation of RNA therapeutics with improved delivery platforms [186,187]. These trials collectively highlight both the promise and the challenges of miRNA-based drugs in oncology [188]. While early programs faced limitations due to immune-related toxicities and delivery inefficiencies, advances in synthetic chemistry, nanoparticle engineering, and tumor-targeted carriers are paving the way for safer and more effective clinical translation [189]. In particular, combining miRNA therapeutics with checkpoint inhibitors or other immunotherapies may unlock synergistic effects, positioning miRNA-based strategies as valuable tools in the evolving landscape of precision immuno-oncology [180,187].

## 4. miRNAs as Biomarkers in Cancer Immunotherapy

### 4.1. Predictive Biomarkers for Immunotherapy Response

Circulating and tissue miRNA signatures have shown strong potential for predicting patient responses to immune checkpoint inhibitors (ICIs) (Table 1), providing non-invasive biomarkers that reflect dynamic tumor–immune interactions [190,191]. In non-small cell lung cancer (NSCLC), higher pretreatment tumor and plasma levels of miR-155 and miR-146a are associated with better responses to anti-PD-1 therapy, with responders exhibiting a 2.5-fold higher median miR-155 expression and a 1.8-fold higher miR-146a level compared to non-responders. Longitudinal monitoring revealed that increasing miR-155 levels during treatment predicts durable clinical benefits and longer progression-free survival [192,193]. In melanoma patients, exosomal miR-21 levels measured before ICI treatment inversely relate to objective response rates. Patients in the lowest quartile of exosomal miR-21 have a 60% response rate, compared to 20% in the highest quartile, suggesting that tumor-derived exosomal miR-21 may act as a marker of an immunosuppressive microenvironment that impairs checkpoint blockade effectiveness [194,195]. These results were reinforced at ASCO 2025, where a prospective multi-center study confirmed that a four-miRNA plasma panel—including miR-155, miR-146a, miR-21, and miR-126—predicts response to anti-PD-1/PD-L1 therapy across 200 patients with melanoma, NSCLC, and renal cell carcinoma, with an area under the receiver operating characteristic curve of 0.87 for distinguishing responders from non-responders [196].

### 4.2. Biomarkers for Immune-Related Adverse Events

Distinct differential miRNA expression profiles have emerged as early indicators of immune-related adverse events (irAEs), enabling preemptive intervention and personalized immune checkpoint inhibitor (ICI) dosing [123]. In patients receiving anti-PD-1 therapy, increases in circulating miR-122 and miR-206 within two weeks of starting treatment predict severe irAEs—including grade ≥ 3 colitis and hepatitis—with 85% sensitivity and 78% specificity, often preceding clinical symptom onset by a median of 10 days and correlating with cytokine storm markers such as IL-6 and TNF-α [205]. Mechanistically, miR-122 influences hepatocyte innate immune responses by targeting TLR4’s adaptor MyD88, while miR-206 controls skeletal muscle–derived IL-6 production, together contributing to systemic inflammatory amplification that underpins severe irAEs [206]. Using miRNA-guided monitoring has allowed early initiation of immunosuppressive therapy in high-risk patients, reducing irAE-related hospitalizations by 40% in pilot cohorts and supporting personalized dosing strategies that balance tumor suppression with safety [195].

### 4.3. AI-Driven miRNA Biomarker Discovery

Artificial intelligence and multi-omics integration have transformed miRNA biomarker discovery, allowing for detailed identification of predictive signatures within complex tumor microenvironments [207,208,209]. Machine learning algorithms trained on both bulk and single-cell RNA sequencing datasets have identified TME-specific miRNA modules, such as a macrophage-enriched miR-21/miR-146 b axis and a dendritic cell–specific miR-155/miR-29 signature, which can classify responders to ICI therapy with over 90% accuracy in cross-validation studies [210,211]. Integrating proteomic and genomic data further enhances biomarker panels by connecting miRNA expression to downstream protein networks and mutation landscapes, supporting the development of integrated predictive models that outperform single-modal biomarkers [212,213]. Recent advances in artificial intelligence have led to the creation of specialized platforms that speed up miRNA biomarker discovery by combining multi-omics data and modeling complex molecular interactions [214]. For example, STmiR uses an XGBoost framework (Table 2) to combine bulk transcriptomic data from TCGA and CCLE with spatial transcriptomics profiles, accurately predicting miRNA activity within specific tissue regions and identifying conserved and cell-type–specific regulators across various cancers [207,215]. JointSyn employs a dual-view deep learning architecture that encodes small-molecule chemical descriptors and cell-line molecular signatures simultaneously, delivering strong predictions of personalized miRNA–drug synergies with R^2^ values around 0.78 and Pearson correlations close to 0.89 [211,216]. SMTRI uses convolutional neural networks to transform miRNA–mRNA duplex secondary structures into simplified numerical formats, enabling rapid in silico screening of small molecules that disrupt specific miRNA–mRNA interactions [208]. Lastly, sChemNET applies graph-based deep learning to learn complex, non-linear relationships between chemical structures and miRNA sequences, facilitating the discovery of new bioactive compounds capable of modulating miRNA function across various cancer types [209]. Collectively, these AI-powered platforms offer powerful tools for identifying predictive miRNA biomarkers and designing combination therapies with remarkable speed and accuracy.

Despite its transformative potential, AI-driven miRNA biomarker discovery faces several challenges. First, heterogeneous data quality and batch effects across different sequencing platforms can introduce technical variability that confounds model training and decreases reproducibility [214,217]. Second, machine learning models trained on limited sample sizes risk overfitting and may not generalize well to independent cohorts, highlighting the need for large, well-annotated datasets and rigorous cross-validation [210,213]. Third, single-cell RNA sequencing data suffer from dropout events and shallow miRNA coverage, which limits the reliable detection of low-abundance miRNAs and makes cell-type–specific signature identification more difficult [218,219]. Fourth, integrating multi-omics layers—including transcriptomics, proteomics, and epigenomics—requires advanced computational frameworks; misalignment between modalities can obscure true biological signals and increase false-positive rates [220,221]. Finally, the “black box” nature of many AI algorithms hampers mechanistic interpretability, making it hard to derive actionable insights or validate candidate biomarkers experimentally without extensive downstream functional studies [208,222].

## 5. Challenges and Future Directions

MiRNA-based immunotherapy faces the inherent challenge of context specificity, as the same miRNA can have different functions depending on tumor type, microenvironment, and cellular state [223,224]. For example, miR-155 promotes pro-inflammatory M1 macrophage polarization and Th1 T-cell responses in bacterial infection models but promotes M2-like immunosuppressive phenotypes in certain tumor contexts through the miR-155/SHIP1 axis [225]. miR-210 acts as a hypoxamir under low-oxygen conditions to enhance immune evasion, whereas miR-200c functions as a tumor suppressor under normal oxygen conditions to reverse metastatic phenotypes [4]. This functional flexibility complicates predicting therapeutic outcomes and requires careful profiling of miRNA roles in each cancer type [226]. Delivery barriers are also a critical hurdle: unmodified miRNAs are rapidly degraded in circulation, and synthetic carriers risk off-target effects and activating innate immunity [227,228]. Although lipid nanoparticles and exosome-based vehicles improve stability and uptake, active targeting strategies like pH-sensitive nanoparticles and aptamer-guided systems are necessary to ensure precise tumor targeting while avoiding toxic side effects [229,230]. Manufacturing scalability and regulatory standardization of complex nanocarriers further hinder clinical application [231]. Tumors also develop adaptive resistance mechanisms that can undermine miRNA therapies by upregulating compensatory oncomiRs, altering RNA-binding proteins, or remodeling extracellular vesicle landscapes to sequester therapeutic miRNAs [227]. Overcoming these adaptive resistance pathways will require combination strategies that integrate miRNA modulation with immune checkpoint inhibitors, mRNA vaccines, CAR T-cells, and targeted small molecules to achieve lasting antitumor responses across diverse tumor ecosystems [4].

Emerging technologies provide promising solutions. Single-cell and spatial transcriptomics enable high-resolution mapping of miRNA activity across different cell types and niches, uncovering cell-type-specific miRNA regulators that guide precision-targeted therapies [232,233]. CRISPR-based miRNA editing permits direct genomic manipulation of oncogenic or tumor-suppressive miRNAs in situ, with Cas9, Cas12a, and Cas13 platforms offering versatile options for multiplexed miRNA reprogramming [234,235]. AI-driven approaches accelerate the discovery of miRNA targets and synergistic drug combinations by modeling complex miRNA–mRNA–protein interactions and predicting optimal therapeutic pairings from multi-omics datasets [22,236].

Looking ahead, expanding miRNA research into rare cancers such as sarcomas and neuroendocrine tumors is crucial due to their unmet therapeutic needs. Early data suggest that miR-34a and neuroendocrine-specific miRNAs (e.g., miR-375, miR-7) control important oncogenic and hormonal pathways, providing new diagnostic and treatment opportunities. Combining miRNA mimics or inhibitors with immune checkpoint inhibitors, vaccines, and CAR T-cells should be a priority in preclinical and early-stage clinical trials to find the best combinations and dosing strategies. Additionally, moving from research to clinical practice requires standardized protocols for validating miRNA biomarkers, including amplification-free assays and AI-enhanced diagnostics, to support regulatory approval and adoption in personalized cancer care. By overcoming these challenges through collaboration across disciplines, developing advanced delivery systems, and designing innovative trials, miRNA-based immunotherapy can emerge as a cornerstone of precision cancer treatment.

## 6. Conclusions

MicroRNAs are at the intersection of tumor biology and immune regulation, making them influential modulators of cancer immunotherapy. Growing evidence highlights their ability to regulate immune checkpoints, reprogram the tumor microenvironment, and act as predictive biomarkers of treatment response. Although early clinical trials of miRNA therapeutics faced limitations due to immune-related toxicities and delivery challenges, ongoing innovations in nanoparticle carriers, exosome-based delivery, and chemically stabilized RNA analogs are addressing these issues. Additionally, combining miRNA profiling with multi-omics strategies and artificial intelligence is expected to improve patient stratification and speed up biomarker discovery. Looking forward, combined approaches that use miRNA-based therapies alongside immune checkpoint inhibitors, CAR-T cells, or cancer vaccines show great potential. Continued research and clinical development will reveal whether miRNA-directed treatments can become effective next-generation tools in precision immuno-oncology.

## Figures and Tables

**Table 1:** Key microRNAs demonstrating mechanistic roles in cancer immunotherapy, summarizing their targets, functional effects and therapeutic contexts.

miRNA	Molecular Target(s)	Functional Effect	Cancer Type	Therapeutic Context	References
miR-138-5p	PD-L1 (CD274)	↓ PD-L1 expression; enhances CD8^+^ T-cell activity	Non-small cell lung cancer	Synergy with anti-PD-1; aerosol delivery	[18,122,197]
miR-34a	PD-L1, BCL2, MET	↓ checkpoint ligands & oncogenes; ↑ apoptosis	Triple-negative breast cancer	Combined with anti-PD-1; lipid nanoparticles	[67,122,198]
miR-200c	ZEB1, E-cadherin (indirect)	Restores epithelial phenotype; ↑ antigen presentation	Lung adenocarcinoma	Synergy with anti-CTLA-4; exosome-mimetic nanovesicles	[199,200]
miR-21	PTEN	Restores tumor suppressor; ↓ PD-L1 expression	Microsatellite-stable colorectal cancer	Anti-miR-21 + anti-PD-1 combination	[47]
miR-155	SOCS-1, c-Fos, Arg-2, Jarid2	↑ DC maturation; ↑ IL-12p70; ↑ Th1 polarization	Melanoma, breast cancer (vaccine models)	miR-155-enriched DC vaccines; mRNA vaccine adjuvant	[41]
miR-17~92	PTEN (indirect via PI3K-AKT)	↑ CD8^+^ T-cell proliferation; ↑ memory formation	Glioblastoma	miR-17~92-enhanced CAR T-cell persistence	[176]
miR-146a	TRAF6, RIPK2, PTGES2	↓ inflammation; ↓ angiogenesis; ↓ tumor-associated fibroblasts	Colorectal cancer	Exosome-mediated delivery; anti-TAF strategy	[201]
miR-145	TGFβRII, IGF1R	Reprograms macrophages to M1 phenotype	Lung cancer (metastasis models)	Nanoparticle delivery; combined with IGF1R inhibitors	[202]
miR-122	KIR (putative), MyD88	Modulates NK activation; ↓ cytokine storm markers	Hepatocellular carcinoma	miR-122 replacement + checkpoint blockade	[203]
miR-206	IL-6 (indirect via muscle source)	Predicts irAE risk; modulates cytokine storms	NSCLC (anti-PD-1 therapy)	Biomarker for irAE monitoring	[204]

↓—Downregulation. ↑—Upregulation.

**Table 2:** AI-driven platforms for miRNA biomarker discovery and therapeutic synergy prediction.

Platform	Methodology	Integrated Data	Application	Performance Metrics	References
STmiR	XGBoost model	Bulk RNA-seq (TCGA, CCLE) + spatial transcriptomics	Predicts spatially resolved miRNA activity and identifies conserved and cell-type–specific regulators	Spearman’s ρ > 0.8 across multiple cancer types	[207,215]
JointSyn	Dual-view deep learning	Small-molecule chemical descriptors + cell-line molecular profiles	Predicts personalized miRNA–drug synergy combinations	R^2^ ≈ 0.78; Pearson r ≈ 0.89	[211,216]
SMTRI	Convolutional neural network	Simplified numerical representations of miRNA–mRNA duplex secondary structures	Screens small molecules targeting specific miRNA–mRNA interactions	High predictive accuracy (AUROC > 0.85)	[208]
sChemNET	Graph-based deep learning	Small-molecule structural features + miRNA sequence data	De novo prediction of bioactive compounds modulating miRNA function	Cross-validated accuracy > 0.9	[209]

## Data Availability

This narrative review synthesizes information from previously published studies, which are appropriately cited within the manuscript. No new data was generated or analyzed in this study. Therefore, data sharing is not applicable.

## Abbreviations

The following abbreviations are used in this manuscript:

3′UTRs	3′ untranslated regions
AGO2	Argonaute 2
AP-1	Activator protein 1
ASCO	American Society of Clinical Oncology (inferred from context)
AUROC	Area under the receiver operating characteristic curve
BCL2	B-cell lymphoma 2
BMI-1	B lymphoma Mo-MLV insertion region 1 homolog
CAR	Chimeric antigen receptor
CCLE	Cancer Cell Line Encyclopedia
CCR7	C-C chemokine receptor type 7
CD274	Cluster of differentiation 274 (also known as PD-L1)
CD40	Cluster of differentiation 40
CD47	Cluster of differentiation 47
CD80	Cluster of differentiation 80
CD86	Cluster of differentiation 86
CSF1R	Colony-stimulating factor 1 receptor
CTLA-4	Cytotoxic T-lymphocyte-associated protein 4
DC	Dendritic cell
DLin-MC3-DMA	2,2-dilinoleyl-4-(2-dimethylaminoethyl)-dioxolane
DODMA	1,2-dioleyloxy-3-dimethylaminopropane
DSS	Dextran sulfate sodium (in colitis models)
EPR	Enhanced permeability and retention
ESCRT	Endosomal sorting complex required for transport
EV	Extracellular vesicle
FA/PS/miR-34a/PS@NDs	Folic acid/protamine/miR-34a/protamine@nanodiamond nanohybrids
HEXPO	Enhanced plant-derived vesicles for xenograft penetration and oncolytic effect
HIF-1α	Hypoxia-inducible factor 1-alpha
HOXD10	Homeobox D10
ICI	Immune checkpoint inhibitor
IFN-γ	Interferon gamma
IGF1R	Insulin-like growth factor 1 receptor
IL-12	Interleukin-12
IL-12p70	Interleukin-12 p70 subunit
IL-17	Interleukin-17
IL-1β	Interleukin-1 beta
IL-6	Interleukin-6
IL-23	Interleukin-23
INT-1B3	Investigational miR-193a-3p mimic (lipid nanoparticle-formulated)
irAEs	Immune-related adverse events
JAK/STAT	Janus kinase/signal transducers and activators of transcription
Jarid2	Jumonji and AT-rich interaction domain containing 2
KIR	Killer immunoglobulin-like receptor
KLF3	Krüppel-like factor 3
KLRG1	Killer cell lectin-like receptor G1
LNP	Lipid nanoparticle
LPS	Lipopolysaccharide
M1	Pro-inflammatory macrophage phenotype
M2	Immunosuppressive macrophage phenotype
MET	Mesenchymal–epithelial transition factor
MHC	Major histocompatibility complex
MICB	MHC class I polypeptide-related sequence B
MN-anti-miR10b	Magnetic nanoparticle-conjugated anti-miR-10b
MRG-106	Cobomarsen (anti-miR-155)
MRX34	Liposomal miR-34a mimic
MSC-EV	Mesenchymal stem cell-derived extracellular vesicle
MyD88	Myeloid differentiation primary response 88
NF-κB	Nuclear factor kappa-light-chain-enhancer of activated B cells
NFAT	Nuclear factor of activated T-cells
NK	Natural killer
NKG2D	Natural killer group 2D
NSCLC	Non-small cell lung cancer
Oct4	Octamer-binding transcription factor 4
PAMP	Pathogen-associated molecular pattern
PD-1	Programmed cell death protein 1
PD-L1	Programmed death-ligand 1
PDGFRA	Platelet-derived growth factor receptor alpha
PDGFRα	Platelet-derived growth factor receptor alpha
PDGFRβ	Platelet-derived growth factor receptor beta
Phf19	PHD finger protein 19
PI3K/AKT	Phosphatidylinositol 3-kinase/protein kinase B
PNET	Pancreatic neuroendocrine tumor
PRC2	Polycomb repressor complex 2
PTEN	Phosphatase and tensin homolog
PTGES2	Prostaglandin E synthase 2
RISC	RNA-induced silencing complex
RIPK2	Receptor-interacting protein kinase 2
RNA-seq	RNA sequencing
SELEX	Systematic evolution of ligands by exponential enrichment
SERPINE1	Serpin family E member 1
SHIP1	SH2 domain-containing inositol phosphatase 1
SIRT1	Sirtuin 1
SMAD	Mothers against decapentaplegic homolog
SOCS-1	Suppressor of cytokine signaling 1
STAT3	Signal transducer and activator of transcription 3
SYT1	Synaptotagmin I
TAF	Tumor-associated fibroblast
TCGA	The Cancer Genome Atlas
TGF-β	Transforming growth factor beta
Th1	T helper 1
Th2	T helper 2
TLR	Toll-like receptor
TME	Tumor microenvironment
TNF-α	Tumor necrosis factor alpha
TRAF6	TNF receptor-associated factor 6
TβRII	Transforming growth factor beta receptor II
UbiC	Ubiquitin C
ZEB1	Zinc finger E-box-binding homeobox 1
